# Animal Chemistry with Reference to the Physiology and Pathology of Man

**Published:** 1846-09

**Authors:** 


### REVIEWS.

---

ART. V.—*Animal Chemistry with reference to the Physiology and Pathology of Man.* By Dr. J. FRANZ SIMON, Fellow of the Society for the advancement of Physiological Chemistry. etc., etc. Translated and edited by GEORGE E. DAY, M.A.&L.M., Cantab.; Licentiate of the Royal College of Physicians. Philadelphia: Lea & Blanchard. 1846.

On a former occasion, in a notice of the first part of this work, we expressed the high estimate in which we held it, and deem any further commendation of it at this time unnecessary. The second part which has just been published, and a copy of which has been sent to us by the publishers, completes the work, making a volume of more than seven hundred pages. The size of this treatise will be objectionable with many readers. It will require some resolution to sit down to a volume of such dimensions made up in great part of tables of analysis; but if not many will thus engage in its regular perusal, few, we should suppose, would be willing to be without it as a work for reference. The part just issued contains ten chapters and an appendix, which treat of the secretions of the chylopoietic viscera and the theory of digestion; milk; secretions of the mucous membranes; secretions of the external skin; the urine; secretions of the lachrymal, meibomian and ceruminous glands; secretions and fluids of the generative organs; the intestinal excretion; the component parts of the animal body; solid morbid products; fluid products of disease. Besides these, many other subjects are presented in the appendix, from which it will be seen that the volume covers the whole chemistry of man.

The saliva is an insipid fluid; destitute of odor, and of very feeble chemical reaction, which would incline one to suppose that it could not possess much scientific interest; but it appears on closer examination to be a somewhat composite fluid. The amount of solid residue in it is small, but small as it is, it affords on analysis "fat, ptyalin, water-extract, spirit-extract, a little albumen, certain salts, and a trace of sulpho-cyanogen." The saliva, according to Berzelius and Müller, takes no active part in the process of digestion. Liebig supposes that one of its offices is to carry oxygen into the stomach, which takes part in reducing the food to the state of chyme. The offices performed by saliva in the animal economy are stated by Dr. Wright to be:—"*Active.* 1. To stimulate the stomach and excite it to activity by contact. 2. To aid the digestion of food by a specific action upon the food itself. 3. To neutralize any undue acidity in the stomach by supplying a proportionate alkali. *Passive.* 1. To assist the sense of taste. 2. To favor the expression of the voice. 3. To clear the mucous membrane of the mouth, and to moderate the thirst." Saliva seems to possess the power of converting boiled starch into sugar, and in this way has some agency in digestion.

The bile is a much more composite fluid, and one which has been a great deal studied. The interest of this secretion to the medical man is greater than that of most others of the animal economy, because of its intimate bearing upon health and disease. In all ages of medicine the bile has been regarded as the secretion especially involved in a long train of human infirmities both of the body and the mind. Horace complains of the effects of his "difficile bilo," and the maladies which have been ascribed to the presence of "black bile" in the body are almost without number. We are attaining to something more like precise knowledge respecting the uses of this secretion, and have, at least, become persuaded that half the disorders of the human body do not arise from biliary derangement. We have come, pretty generally, to regard the bile as something more than an excretion; or as having something more to do than merely be "pumped" out of the liver by purgatives. Dr. Simon says:

"Bilin and urea can hardly be regarded as simultaneous products of the metamorphic action of the blood; for while I have detected small quantities of urea in the blood of a healthy calf, I have never been able to recognise the least trace of bilin or of bile-pigment. Hence, while urea is produced not only in the kidneys but in other parts of the system, bilin seems to be produced and secreted only in the liver."

What are its precise agencies in the complex operations of the animal body physiologists are far from being satisfied; and indeed chemists are very far from being agreed as to what is its precise composition. It is by no means an easy matter to arrive at satisfactory conclusions on this subject. Remarks Dr. Simon:—

"An accurate quantitative determination of the most important ingredients of the bile, although difficult, is by no means impracticable. It is, however very uncertain whether the result of the analysis would afford any insight into the true character of that changeable secretion. From the latest researches of Berzelius, it appears that the bilin is so unstable a compound, that it is hardly possible to obtain bile in the condition in which it is secreted by the liver, or as it exists in the gall-bladder; for when bile is left to itself, and much more when it is acted on by heat and other more or less energetic agents, the bilin undergoes a series of metamorphoses by which fellinic, cholinic, and very probably also cholanic and fellanic acids are produced. The biliary secretion, as it exists in the liver, may be regarded as pure bilin mixed with biliverdin and fats; the bilin probably commences its metamorphoses in the gall-bladder, and continues them in its passage onwards into the intestinal canal. If fellinic and cholinic acids are formed in the gall-bladder, then the presence of the two bilifellinic acids in fresh bile may be at once assumed, since they are only to be regarded as combinations of the former with different proportions of bilin. It is not by any means probable that cholic acid exists in fresh bile, and the presence of dyslysin and taurin may be positively denied; consequently, the biliary resin, the mixture of fellinic and cholinic acids and dyslysin does not pre-exist in the bile."

In a note the editor adds the following remarks concerning the destination of the bile:

"That the bile is not merely an excrementitious fluid, intended to remove effete matter from the blood, but that it is a secretion essential to the animal economy, was rendered almost certain by the experiments of Berzelius, Theyer, and Schlosser, which showed that the human fæces contained much too small a quantity of a substance resembling bile to justify the idea that it is evacuated in this manner. A further proof that the bile is absorbed and not excreted, is afforded by an examination made by Enderlin, of the ash yielded by the contents of the different portions of the intestinal canal of a hare. He found that the ash from the contents of the duodenum alone effervesced on the addition of an acid, thus showing that the choleate of soda (which yields the carbonate by incineration,) is absorbed before reaching the jejunum. Schwann has recently established this opinion beyond a doubt, by a series of well-devised experiments on dogs. He tied the ductus communis choledochus, and at the same time formed a fistulous opening in the gall-bladder, by which the bile escaped externally. His most important conclusions are—

"1st. That when the bile does not get into the bowel, its absence is generally perceptible in dogs, about the third day, by a marked diminution in weight.

"2dly. That unless the channel for the conveyance of the bile to the duodenum be re-established, symptoms of deficient nutrition, wasting, debility, &c., ensue, and death is the ultimate consequence."

The section on the *gastric juice, digestion, and the chyme,* is one of great interest, and has been carefully elaborated. What is gastric juice? is a question often propounded, and never yet satisfactorily answered, as appears from the following paragraph:

"The gastric juice has been examined by numerous chemists, in consequence of the importance attributed to it in the process of digestion. There have been found in it free acids, a considerable amount of salts, and certain indefinite animal substances, which were known at the period to which we refer as osmazome or salivary matter. Experiments on artificial digestion have thrown much light on the nature of the gastric juice. Eberle proved that artificially-formed gastric juice does not thoroughly dissolve food, unless a small quantity of gastric mucus, or a portion of the mucous membrane of the stomach be added to it. On the strength of this discovery, Müller and Schwann instituted a series of experiments, from which Schwann was led to conclude that the gastric juice contains a peculiar substance, which, co-operating with an acid, possesses the property of dissolving substances insoluble in mere water, or in a mixture of extractive matters, salts, and a little acid, as for instance, fibrin, coagulated albumen or casein. To this somewhat problematic substance he gives the name of *pepsin:* Wassmann and Pappenheim have endeavored to isolate it."

Since Dr. Simon's labors ceased, the process of digestion has called forth several investigators, and elicited a multitude of experments, to some of which we have heretofore alluded. Blondlot's treatise on digestion promises to take a high rank among the works of which this difficult subject has been so fruitful. It is quoted largely by Dr. Day in the volume before us. According to this writer—

"M. Blondlot believes that the digestive property of gastric juice depends, not on its obvious chemical constitution, but upon a peculiar organic principle. If exposed to a temperature of 104° to 122° F., or higher, it loses entirely and irrevocably its digestive powers, although to all appearance, and even as to its composition, as made known by analysis, it remains unchanged. With the exclusion of the air, gastric juice may be kept for two years without loss of activity; but with the free access of air, it putrifies in five or six days, although the chyme which it forms from nitrogenous organic substances may be preserved for two or three months without change. The precipitation of all the lime it contains does not affect its activity, nor are its chlorides indispensable, but whatever acts upon its organic constituents, (heat, strong alcohol, or strong acids,) or which removes them, (such as animal charcoal, chlorine, tannic acid, or acetate of lead,) destroys all its digestive properties.

"M. Blondlot also shows—*a.* That coagulated albumen resists the action of the gastric juice only from its compact form. When coagulated in very small particles, as the white of an egg beaten into a froth and poured into boiling water, it is digested as quickly as soft fibrin. *b.* That the action of the stomach in coagulating milk is not due to its digestive principle solely, but to its acid, which acts like lactic acid. *c.* That the effect of the gastric fluid upon bones, whether entire or not, is to disintegrate them slowly, beginning at the surface, and to reduce the earthy matter into a fine chalky powder, but without dissolving or decomposing it. The earthy matter not being dissolved, proves that no hydrochloric acid has acted upon it; it is all discharged with the fæces."

Since the publication of Blondlot's work, Bernard and Barreswil, two French chemists, have published the result of their experiments on the gastric juice. They assume that this fluid owes its digestive properties to the union of two principles—an acid, and a peculiar organic matter destructible by heat. Their views are set forth in the following passages:

"The principal fact which has been adduced to prove that the acid reaction is owing to the presence of biphosphate of lime is, that it may be treated with carbonate of lime without effervescence. Our experiments show that this arises from the dilution of the acid, which allows the carbonic acid to be dissolved as it is formed. When, therefore, the gastric juice is concentrated, it causes a considerable effervescence with chalk. Moreover, gastric juice dissolves neutral phosphate of lime, whilst this salt is entirely insoluble in a solution of the biphosphate. On distilling gastric juice, the first distillate exhibits no acid reaction. If a mere trace of acetic acid or acetate of soda is added previous to distillation, it gives no acid reaction; the normal acid is not therefore acetic. This also appeared, at first sight, to prove it could not be hydrochloric acid; but on distilling water rendered slightly acid by hydrochloric acid, nothing passes over at first but pure water, the acid not distilling until the end of the operation. On distilling gastric juice, a neutral limpid liquor passes over, which is not precipitated with nitrate of silver; when about four-fifths has distilled over, the distillate is perceptibly acid, nevertheless, it does not render a solution of nitrate of silver turbid; but at the end, and when only a few drops of the gastric juice remain in the retort, an acid liquid passes over which precipitates salts of silver; this is, doubtless, hydrochloric acid. Does this acid exist free in gastric juice, or has a chloride been decomposed in this operation? When the least trace of oxalic acid is added to gastric juice which we know contains lime, a turbidity is produced from the formation of an insoluble oxalate of lime; but if to water acidified with 2000ths of its amount of hydrochloric acid, and containing chloride of lime, the same reagent be added, no turbidity ensues. This clearly proves that hydrochloric acid exists as a chloride in the gastric juice, and not in a free state.

"When concentrated by evaporation, gastric juice is highly acid, effervescing with chalk, and not losing its acid reaction in the presence of an excess of the chalk. This proves the presence of phosphoric acid. On saturating the acid with lime and oxide of zinc, and filtering the solution, the neutral filtrate contains both zinc and lime, therefore phosphoric acid is not the only free acid in the juice. What is the acid combined with the zinc and lime in the filtered solution? It is one which, as we have seen, passes over at the end of the distillation, and does not precipitate salts of silver. These characters belong to lactic acid. On distilling water slightly acidulated with lactic acid, a small quantity of chloride of sodium being added, we obtain a fluid analogous to gastric juice; first, pure water passes over, then an acid which does not precipitate salts of silver, and the last drops carry over hydrochloric acid. So that it is evident that the presence of hydrochloric acid in the last product of distillation of the gastric juice is owing to the decomposition of the chlorides by lactic acid."

What the acid in the stomach is which seems to be concerned in digestion, the same authors attempt to determine. They believe it is not hydrochloric or acetic acid, nor indeed any particular acid; for although they always found the lactic present in the gastric fluids, they do not believe that the digestive powers of the gastric juice are owing to this acid. They conclude, as the result of their researches, that if an acid reaction be indispensable at all, other acids may take the place of the lactic. The digestive power of the fluids of the stomach they hold to depend upon the presence of a peculiar organic matter, the properties of which are as follows:—

"It is precipitated and destroyed at a temperature of 190°. One of the most remarkable of its properties is that its digestive powers vary according to the medium in which it is contained. In the gastric juice, which is acid, it dissolves nitrogenous matters, such as fibrin, gluten, and albumen; but exerts no action on baked starch; but if the gastric juice is rendered alkaline by the addition of a little carbonate of soda, it rapidly dissolves the starch, and no longer possesses the power of acting on the nitrogenous matters. As these physiological properties are exactly those of saliva and the pancreatic fluid, it became an interesting point to ascertain if a change in the reaction of these fluids would cause a corresponding variation in their solvent power. This was found to be the case; on acidulating these naturally alkaline fluids, their ordinary mode of action was inverted, and they were enabled to dissolve nitrogenous matters, while their capability of dissolving starch was lost. From numerous and varied experiments they believe that one and the same organic principle (the agent of digestion) exists in the gastric juice, the pancreatic fluid, and the saliva, and that its physiological action varies according to the acid or alkaline nature of the fluid in which it occurs."

Our notions concerning the process of digestion are still in a most unsettled and crude state; we have, indeed, very little knowledge of the changes which the different elements of food undergo in becoming what we style chyme. It is embraced in the following summary of our author:

"1. Albumen is dissolved and chemically changed. This observation was made by Eberle, and has been confirmed by Mûller, Schwann, and others. The digested albumen no longer coagulates at the boiling point; it is stated to have been changed into osmazome and salivary matter, (a vague statement requiring further proof,) and according to Schwann, into a third albuminous principle, which is thrown down by carbonate of soda, and in that condition is insoluble in water and spirit, soluble in muriate of soda, and not precipitable by acetate of lead or alcohol, but copiously by nitric acid and bichloride of mercury, and partially by ferrocyanide of potassium and tannic acid.

"2. Coagulated casein is partially converted by artificial digestion into albumen; soluble casein becomes coagulated when submitted to the action of a solution of sugar of milk and pepsin, but not when acted on by the pepsin alone.

"3. Fibrin is rapidly dissolved, and, from the experiments of Tiedemann and Gmelin, appears to be partially converted into albumen.

"4. Glutin becomes so changed by artificial digestion, that it loses its property of gelatinizing, and can no longer be precipitated by chlorine.

"5. Sugar of milk, when submitted for a sufficient time to the action of pepsin, becomes completely converted into lactic acid. This fact has been established by Fremy and myself.

"6. Starch is partially converted into sugar. (Tiedemann and Gmelin.)

"7. The fluid found in the stomach of a horse, fed with oats, contained butyric acid, a resin, a substance resembling extract of flesh, salivary matter, and albumen."

The celebrated William Hunter was among those who held the digestive process to be one essentially vital, and in addressing his class on the subject he is reported to have used the following language:—"Physiologists will have it, gentlemen, that the stomach is a mill; others that it is a fermenting vat; others again that it is a stew pan; but in my view of the matter, it is neither a mill, a fermenting vat, nor a stew pan, but a *stomach*, gentlemen, a stomach." But this lucid view of the matter has not been universally adopted. There are those who still hold that a stomach is not quite indispensable to the business of digestion. Dr. Simon, from the following, seems to be of that number.

"From recent experiments on digestion, we know that alimentary substances are dissolved as rapidly in an artificial digestive fluid, consisting of pepsin and properly diluted muriatic acid, as they are in the gastric juice itself. Hence we are justified in the conclusion that pepsin, the free acid, and a suitable temperature, are the principal agents in gastric digestion, and that the motions of the stomach are chiefly with the view of promoting the due admixture of the food with the secreted fluid, and of propelling it towards the pylorus, through which it must pass in order to enter the duodenum. It is impossible to state with certainty whether the pepsin and free acids dissolve and modify the food through a catalytic influence, or whether they enter into any chemical combination with it, the products of these combinations being the dissolved and changed matter. If, however, the conversion of sugar of milk into lactic acid is explained by the catalytic action of the pepsin, we may fairly conclude that the pepsin exerts a similar influence on other substances, if no facts to the contrary present themselves. Hünefeld is inclined to attribute considerable influence in digestion to the ammoniacal salts of the gastric juice, in consequence of having observed that under certain conditions fibrin is readily soluble in the muriate or lactate of ammonia, especially when free lactic acid is also present."

It has always appeared to us somewhat inconsistent in the advocates of the vital theory of digestion to insist upon the power of the gastric juice to digest the stomach after death, and at the same time deny that it can digest other matters by virtue of its mere chemical attributes. If it can dissolve the coats of the stomach when the principle of life is no longer present to protect them, why not dissolve the food in the stomach independently of the vital principle? Vitality is most clearly out of the question in one case; why suppose its presence and co-operation necessary in the other? Digestion, where the stomach is the subject and not the agent, is chemical, beyond the possibility of a cavil; why not allow, then, that it is chemical also in all the cases where, not the stomach, but its aliment, undergoes the change? It is not philosophical to multiply causes. If chemistry is adequate to the process in one case, we see no room to question that chemical action is present in all.

We do not propose to follow the author through all his analyses, and shall therefore pass unnoticed many chapters which seem of subordinate importance. The perspirable matter is one of the secretions which the physician never overlooks, and all that can be known respecting it has interest for him. Dr. Simon has the following observations in regard to morbid sweat.

1. *The quantity of the sweat is sometimes increased in an extraordinary degree.*

"Thus critical sweats are usually very abundant, continuous, and watery, in intermittent fevers, in rheumatic affections, and in colliquative disorders.

"2. *The quality of the sweat is changed.*

"*a.* The sweat may be distinguished by a peculiar odor. The sweat of persons with the itch is said to have a mouldy odor, while that of syphilitic patients is said to smell sweet. The sweat of rheumatic and gouty persons has an acid smell, while in putrid fever and scurvy, it has a putrid odor; in jaundice it is said to resemble musk in its smell. In Stark's General Pathology, (p. 1126), we find it stated that the odor of the sweat in scrofula resembles that of sour beer, while in intermittent fever it smells like fresh baked brown bread. The determination of odors is, however, very subjective, and (with a few exceptions) it is more than probable that different observers would detect different resemblances.

"*b.* Some of the normal constituents may be abnormally increased.

"1st. *The free acid of the sweat may be increased.* Lactic acid, which is the ordinary free acid, is usually increased in cases of rheumatism and gout; the sweat in these diseases has a strong acid reaction. When there is also an acid odor acetic acid is present. Prout has found free acetic acid in the sweat of a person suffering from hectic fever. After an attack of acute rheumatism, the joints of the feet remained swelled, for which potash baths were ordered. These baths, in the course of three weeks, brought on an attack of eczema, extending as high as the knee. The sweat from the feet had then a decided odor of acetic acid, which became more strongly developed when they were sharply rubbed. Anselmino found free acetic acid in the sweat of women during their confinement; and, according to Stark, the quantity of free lactic acid is increased in the sweat during scrofula, rachitis, and certain cutaneous eruptions.

"2d. *The ammonia of the sweat may be increased.* Anselmino found a larger proportion of (free?) ammonia in the sweat after an attack of gout than in any other case. Berend states that the sweat in putrid and typhus fever is ammoniacal; and in nervous diseases(?), according to Nauche, it becomes alkaline. All sweat with a putrid odor probably contains free ammonia.

"3d. *The salts may be increased.* Prout observed that in the case of a man with dropsy the skin became covered with a white saline crust of chloride of sodium, after an abundant perspiration. Anselmino found in the sweat, after a severe attack of gout, more salts than usual. In cases of gouty and urinary concretions, the quantity of phosphate of lime appears to be increased.

"*c.* Abnormal constituents may be present in the sweat.

"1st. *Albumen* has been observed by Anselmino in a critical sweat, which broke out in large quantity one evening over the whole body in a case of febris rheumatica, with severe pains in the joints; on the following day it had disappeared. Stark asserts that albumen may be found in the sweat in gastric, putrid, and hectic diseases, and also on the approach of death, in consequence of the abnormal solution of the solid constituents. I failed in detecting any certain indications of albumen in sweat collected (by means of linen washed with distilled water) from the breast of a person in the colliquative stage of tubercular phthisis.

"2d. *Blood or its constituents.* Voigtel observed an instance of bloody sweat from under the arm of a young man; it appeared after any violent exertion. In scurvy, putrid fever, and typhus icterodes, bloody sweat has likewise been observed.

"3d. *Uric acid* is stated to have been found in the sweat of arthritic persons (Stark). Wolff found that the sweat which had hardened on the forehead into a solid white substance, (in a patient with stone in the bladder), contained uric acid. Urate of soda is likewise stated to have been found in the sweat of persons suffering from gout or stone.

"4th. *Bilin and biliphæin* have been found in the sweat of persons with jaundice, and sometimes in such large quantity as to color the linen yellow, and to communicate a bitter taste to the perspiration. According to Berend, the sweat in febris putrida biliosa likewise contains bile-pigment.

"5th. *Red coloring matter of the urine* (*uroerythrin*) was found by Landerer in sweat from the axilla of a fever patient. A blue coloring matter, doubtless allied to cyanurin, has occasionally been observed in the sweat. Dr. Bleifuss has seen blue sweat from the foot of a patient with disease of the abdomen. Michel has likewise observed it in an hysterical woman and in a hypochondriacal man; it was most marked on the right side of the body. Billard observed a blue sweat on the upper part of the body of a girl.

"6th. *Fat* is stated to occur in colliquative hectic sweats.

"*d.* Substances altogether foreign to the animal organism may be conveyed, through the process of digestion, into the blood, and then occur in the sweat.

"Landerer has observed in his own person that after taking large doses of quinine, the sweat assumed a bitter taste of the drug. The following substances enter into, and have been detected in the sweat; sulphur, mercury, iodine, iodide of potassium, asafœtida, garlic, saffron, olive oil, rhubarb, indigo, prussian blue, and copper. (Stark, General Pathology, p. 1127; Baumgärtner, Elements of Physiology and Therapeutics, p. 486.)

"Many of these statements, regarding the change undergone by the sweat in disease, are fully confirmed; some must, however, still be regarded as doubtful."

A hundred and eighty-two pages of this volume are taken up with the consideration of the urine, one the most complex secretions of the animal body. Still, our author remarks, the relative proportions of its different constituents are not very variable. The following are stated to be the ordinary constituents of healthy human urine: "Urea; uric acid; [hippuric acid;] extractive matters, embracing alcohol-extract, spirit-extract, and water-extract, with their respective constituents; mucus; brown coloring matter of the urine (hæmaphæin); red coloring matter of the urine (uroerythrin); carbonic, lactic, hydrochloric, sulphuric, phosphoric, silicic, and hydrofluoric acids; soda; potash; ammonia; lime; magnesia; and peroxide of iron."

The qualitative analysis of urine is not difficult, many of its elements being easily detected; but some are discovered only by isolating them, which requires a more thorough analysis. The specific gravity, which throws a good deal of light upon the character of the urine, may be accurately and very conveniently determined by the ordinary 1000-grain bottle, which physicians would do well to make a part of their shop-furniture. This bottle is used with convenience in examinations of the blood, the specific gravity of which it is often of consequence to determine.

The urine must vary in its constitution with the nature of the food taken by the individual. It is not the same under a diet of animal matters, as when the food is vegetable. Numberless soluble substances reach the urine from the alimentary canal, and affect its color, taste, and odor, as well as its chemical properties. It is acid under certain circumstances, and under others alkaline. And what is still more curious, it often contains a larger quantity of certain salts than was to have been found in the food of the individual. The sulphates, for example, are frequently found in excess in this secretion, having come from the oxidation of sulphur in the animal economy, sulphuric acid being developed, which, uniting with the bases, affords these salts. Something analogous to this we see going on in nature around us every day, where *sulphuret* of iron becomes the *sulphate* of iron by oxidation in the atmosphere. As this slow combustion of sulphur and iron is attended with the formation of sulphuric acid and a sulphate, so in the animal economy the sulphuret of potassium becomes the sulphate of potash by imbibing an additional dose of oxygen. The following remarks of Dr. Simon on this subject will be read with interest:

"It is clear that the excretion of a salt taken with most of our articles of food, must be entirely dependent on the quantity consumed, and must therefore vary very considerably.

The urine, generally speaking, is deficient in salts during disease: our analyses show that the deficiency occurs at the expense of the chloride of sodium; the sulphates and the phosphates taking only a small part in it. I have analysed urine in typhus which contained a mere trace of chloride of sodium.

"Lecanu observed differences similar to those we have just noticed in alkaline sulphates and phosphates. The quantities excreted in twenty-four hours not only varied in different persons, but also in the same person at different times. From my analyses, and those of Lehmann, it appears probable that a connexion subsists between the quantity of urea and of the sulphates, and possibly of the phosphates likewise; that is to say, the sulphates always increase with the urea, and *vice versa.* I incline, therefore, to the opinion of Berzelius, that at least a portion of the sulphates and phosphates owe their origin to the oxidation of sulphur and phosphorus, previously associated with protein which has become changed during the active metamorphosis of the blood. We do not, however, mean, in making this statement, to deny that the salts are also supplied to the blood by the food, and again separated by the kidneys.

"The five following results, of much importance in physiology, have been deduced from the admirable researches of Lecanu:

"1. The quantity of urea excreted by the same person during equal periods is constant.

"2. The same is the case with respect to the uric acid.

"3. The quantities of urea and uric acid excreted by different persons during equal periods are variable.

"4. The varying amounts of urea excreted during equal periods by different persons, bear a relation to age and sex.

"5. The amount of fixed salts varies in different persons without reference to age or sex. It also varies in the same person during equal periods.

"An observation simultaneously made by Lehmann and myself, appears to me of high physiological import. We have ascertained that the amount of the urea, as well as of the sulphates, is increased by strong bodily exercise. I produced this state by taking such violent exercise for two hours that the pulse continued for some time above 100."

We append his summary in which the leading facts established by the researches of chemists are given in few words:

"To sum up once more: the urine is most abundant in urea, uric acid, and the most important salts, in men in the prime of life; it is less rich in these constituents in women, while the minimum occurs in old men and children. The nature of the food exerts an influence upon the composition of the urine; the amount of urea is increased by an excess of nitrogenous food, and diminished after living on food deficient in nitrogen. Upon a diminution of the quantity of food, the urine becomes deficient in nitrogen, as has been shown by my own experiments and those of Lehmann; but the separation of nitrogenous compounds, as for instance urea, through the urine, occurs even when no food is taken. The urine is most abundant in urea and sulphates after active bodily exercise, in consequence, doubtless, of increased vascular excitement. The quantity of urine discharged in 24 hours, amounts on an average to about 45 ounces. It is more abundant in the prime of life than in old age or childhood, and in the male than in the female sex."

Our author next proceeds to the consideration of the urine as affected by disease. The pathological changes may be reduced to one of the following forms: 1. One or more of the normal constituents of the urine may be in excess. 2. One or more of these may exist in less quantity than in healthy urine. 3. A normal constituent may be absent. 4. Substances not natural to the urine may be present. Something may be inferred respecting the constituents of the urine from its appearance. Simon says—

"I have already observed that the proportion of solid constituents to the water is so very variable, is so dependent upon the vicarious action of the skin and lungs, and upon the quantity of fluid that has been taken into the system, that it is impossible (without taking other facts into consideration) to determine from the urine alone, whether a mere increase or decrease of the solid constituents is due to diseased action. Pale urine, more or less like water, may be fairly considered deficient in solid matters, while a deep brown color is indicative of an abundance of these constituennts.

"The specific gravity, and still more the determination of the solid constituents in the manner which has been already described, will give the required information. The color of the urine is sometimes deceptive, especially the fiery red that occurrs during fevers. Urine of this sort is frequently found to be poorer in solid constituents, and its specific gravity lower than we should have anticipated from its color: it is usually, however, more abundant in uric acid than normal urine."

In hysterical affections, and in nervous disorders generally, the quantity of water in the urine is increased. Becquerel relates a case of a girl who ordinarily secreted daily about thirty-seven ounces of water, but in whom the amount rose to ninety ounces on the occasion of a severe hysterical attack. On the other hand, in inflammatory affections the quantity of water is very much diminished. The author just quoted has seen it fall as low as twelve ounces in twenty-four hours, in patients laboring under the phlegmasiæ. In such cases the urine is dark-colored, of a high specific gravity, and possesses a strong acid reaction. The quantity of urea in such affections is absolutely or relatively increased; the food being reduced it ought to decline, but such is not the case. The waste of the body compensates for the diminution in the quantity of aliment. In diseases marked by a deficiency of blood, the quantity of urea is diminished. Chlorosis may be given as an example, and in typhus Simon found the same to be true. Uric acid varies according to the pathology of the ailment. It is increased in inflammations and the paroxysms of fevers. Our author continues—

"The following pathological conditions lead, according to Becquerel, to an increased quantity of uric acid: fever; great general functional disturbances, such as arise from oppressive dyspnœa in pulmonary emphysema or cardiac disease, acute pain, convulsions, delirium, &c., especially when attended with fever; and diseases of the liver, as hepatitis, cancer, or cirrhosis. The amount of uric acid is diminished in those cases in which there is a deficiency of blood, or where the blood is poor in corpuscles. Becquerel found this to occur in cases of chlorosis and anemia, and in persons in whom the vital juices seemed dried up. The amount of the salts in the urine fluctuates extremely during disease. Generally speaking, we may assume that the quantity of salts decreases in most pathological states of the system; the cases in which the salts increase during disease being very rare. Becquerel states that in those diseases in which the amount of urea is only slightly diminished, the proportion of salts is not materially affected; but that in those cases in which the urea suffers a considerable reduction, the same takes place with regard to the salts. Analyses of inflammatory urine are, however, opposed to this statement, since in these cases the urea sometimes exceeds the normal amount, while the salts are decreased in an extraordinary manner. It is to be regretted that Becquerel has not undertaken an exact quantitative separation of the different salts, as the increase or decrease of the fixed salts collectively is a circumstance of much less importance than the varying relative proportion of the individual compounds."

The physiological average of uric acid, Simon thinks, may be placed at 1·5% of the solid residue; and in the phlogoses he has found it rise as high as 3%, while Becquerel has seen it amount to 5·9% Again, the quantity of extractive matter, which in normal urine amounts to 23·5% of the solid residue, rises in inflammations to 43%, and, on the contrary, the fixed salts decline here from 25 to 12, or precisely one-half. Abstractions of blood change the character of the urine; "it becomes clearer, specifically lighter, and the amount of urea decreases absolutely and relatively."

The urine of lunatics has recently been examined with much care, and it seems that it is characterised by an excessive quantity of ammonia in the form of a carbonate, urate, hydrochlorate, or ammoniaco-magnesian phosphate. In delirium tremens it varies; being now in a normal condition, and again of an inflammatory type; from which we may infer that the disease is not uniform in its character. In bronchitis it is "scanty of a dark-red color, strongly acid, and of a high specific gravity." Becquerel detected albumen in the urine of such cases. In pneumonia the urea is diminished, and the extractive matters, especially the alcohol-extract, are increased. Schonlein, one of the most accurate of observers, has found that "the crisis in pneumonia shows itself in the urine by the secretion becoming turbid and sedimentary; after ten or twelve hours a crystalline micaceous deposit forms, above which the urine becomes clear." Upon which Simon remarks:

"The following instance is strongly confirmatory of Schönlein's opinion. In a case of pneumonia that recently occurred in his own wards, the urine during the height of the inflammatory stage, was dark, very acid, and deposited no sediment; at the period of resolution it became paler and neutral; one morning I found it yellow, neutral, and with a sediment of white crystals visible even to the naked eye. The microscope at once revealed the beautiful shapes assumed by the ammoniaco-magnesian phosphate. I was much struck with the singular relations of the urine itself. It was perfectly neutral; and any acid, even dilute acetic, threw down a white precipitate, which led to the supposition that caseous matter was present; I soon, however, found that this was not the case, for on treating a portion with hydrochloric acid and allowing it to stand for some time, very beautiful, nearly colorless crystals of uric acid were deposited."

It would be interesting to follow our author through his long list of diseases—gastritis. dysentery, pleuritis, hepatitis, nephritis, &c.—and note the changes wrought by each in the condition of the urine; but we must forbear. It may be that we have already extended our notice of his work too far for the taste of many of our readers. If so, while we crave their pardon, we must beg them to remember, that one whose business it is to cater for the palates of others, must be governed somewhat in his choice of subjects by the promptings of his own intellectual appetite. We find "the chemistry of man" to our taste. We like its precision, its condensation, its array of facts. Not a page, not a paragraph, but conveys some truth bearing upon the constitution of the animal frame. Many of these, for the present, may appear to be without any essential import—naked facts, the value of which cannot be perceived; but their utility will become apparent in due season. The field has just been opened; the virgin soil is not yet fairly turned up to the air and the sun; but the harvest is coming on and promises to be a rich one. The chemistry of man deserves a place among our medical studies.

